# Gelatin-Based Scaffolds with Carrageenan and Chitosan for Soft Tissue Regeneration

**DOI:** 10.3390/gels10070426

**Published:** 2024-06-28

**Authors:** Chiara Pasini, Federica Re, Federica Trenta, Domenico Russo, Luciana Sartore

**Affiliations:** 1 Department of Mechanical and Industrial Engineering, University of Brescia, 25123 Brescia, Italy; chiara.pasini1@unibs.it; 2 Blood Diseases and Cell Therapies Unit, Bone Marrow Transplant Unit, “ASST-Spedali Civili” Hospital of Brescia, Department of Clinical and Experimental Sciences, University of Brescia, 25123 Brescia, Italy; federica.re@unibs.it (F.R.); federica.trenta@unibs.it (F.T.); domenico.russo@unibs.it (D.R.); 3 Centro di Ricerca Emato-Oncologico AIL (CREA), “ASST-Spedali Civili” Hospital, 25123 Brescia, Italy

**Keywords:** soft tissue engineering, bioresorbable hydrogels, chitosan, carrageenan, tissue-like viscoelasticity, stress relaxation, biocompatibility, human umbilical cord mesenchymal stem cells

## Abstract

Motivated by the enormous potential of hydrogels in regenerative medicine, new biocompatible gelatin-based hybrid hydrogels were developed through a green process using poly(ethylene glycol) diglycidyl ether as a cross-linking agent, adding carrageenan and chitosan polysaccharides to the network to better mimic the hybrid composition of native extracellular matrix. Overall, the hydrogels show suitable structural stability, high porosity and pore interconnectivity, good swellability, and finally, biocompatibility. Their mechanical behavior, investigated by tensile and compression tests, appears to be characterized by nonlinear elasticity with high compliance values, fast stress-relaxation, and good strain reversibility with no sign of mechanical failure for compressive loading–unloading cycles at relatively high deformation levels of 50%. Degradation tests confirm the hydrogel bioresorbability by gradual hydrolysis, during which the structural integrity of both materials is maintained, while their mechanical behavior becomes more and more compliant. Human Umbilical Cord-derived Mesenchymal Stem Cells (hUC-MSCs) were used to test the hydrogels as potential carriers for cell delivery in tissue engineering. hUC-MSCs cultured inside the hydrogels show a homogenous distribution and maintain their growth and viability for at least 21 days of culture, with an increasing proliferation trend. Hence, this study contributes to a further understanding of the potential use of hybrid hydrogels and hUC-MSCs for a wide range of biomedical applications, particularly in soft tissue engineering.

## 1. Introduction

The success of tissue engineering procedures strongly relies on the development of biomaterial-based scaffolds that can adequately substitute the extracellular matrix (ECM) and at the same time host cells capable of building a new natural ECM. With this purpose, a variety of bioresorbable polymeric scaffolds have been proposed in the literature as temporary substrates for cells [1,2,3]. In particular, hydrogels have emerged for their superior regenerative performance, which is attributable to the close similarity between their hydrophilic macromolecular network and the ECM structure. Like the ECM is composed of many different biomolecules, including fibrous proteins such as collagen and elastin as well as carbohydrates such as those found in proteoglycans, hybrid hydrogels combining different polymer types can be fabricated, aiming at the best assortment of physical, mechanical, chemical, and biological properties for tissue engineering and drug delivery applications [4,5,6,7].

Several hydrogel constituents of a natural origin have been studied due to their excellent biocompatibility, bioresorbability, and bioactive properties. Among these, collagen-derived materials deserve special attention, given the important structural role of collagen fibers in biological tissues. Gelatin, denatured and partially hydrolyzed native collagen, has been widely employed for its good biocompatibility, biodegradability, low cost, and ease of manipulation. While sharing with collagen the presence of cell-binding sites (e.g., arginine-glycine-aspartic acid sequence) and of enzyme-mediated degradation sites, it presents low/no antigenicity. Furthermore, the side groups of its constituent amino acids can be employed to facilitate ionic interactions or crosslinking/grafting reactions with a wide range of naturally occurring polymers and molecules [8].

Many polysaccharides have also been investigated to enhance the bioactive properties of scaffolds, including alginate, chitosan, dextran, carrageenan, and many others [9]. Chitosan, a partially deacetylated derivative of chitin, is particularly appreciated for its cationic surface charge, which creates interactions with negatively charged species and thereby provides it with antibacterial properties and hemostatic activity [10]. Carrageenan is another polysaccharide of interest, derived from several types of red seaweed and consisting of linear and highly sulfated galactans that structurally resemble ECM glycosaminoglycans, leading to improved cell adhesion and proliferation [11,12]. It has been studied for its immunomodulatory, antioxidant, antibacterial, antiviral, and anticoagulant properties [11,13], and is proposed for biomedical applications including controlled drug delivery, cell encapsulation, and wound healing, as well as cartilage and bone tissue engineering [11,12,14,15].

While natural compounds are preferred to improve scaffold bioactivity, they often suffer from poor mechanical properties, uncontrolled degradation kinetics, and low reproducibility; these limitations can be overcome by the addition of synthetic components to the hydrogel network [16]. In this study, poly(ethylene glycol) diglycidyl ether (PEGDGE) was employed as synthetic crosslinker to produce gelatin-based hybrid hydrogels containing carrageenan and chitosan through a green synthesis approach without using any toxic solvent or foaming agent. In particular, two new gelatin-based systems were crosslinked with PEGDGE, one containing carrageenan and the other containing both carrageenan and chitosan.

Moreover, with the aim of exploring the biological potentiality of the developed hydrogel for soft tissue engineering, a novel regenerative model was studied, which employed these hybrid scaffolds in association with Mesenchymal Stromal Cells derived from the Umbilical Cord (hUC-MSCs) supplemented with human platelet lysate (hPL). hUC-MSCs are undifferentiated stem cells with the potential for self-renewal and the capacity to differentiate into several lineages. Since the hUC-MSCs have a high rate of proliferation, immunological tolerance, and lack of tumorigenicity, their use in autologous and allogeneic regenerative medicine applications is widely investigated. MSCs can also have a reparative effect through paracrine signaling by releasing biologically active molecules that affect the cell migration, proliferation, and survival of the surrounding cells [17]. These functions provide therapeutic potential for treating conditions characterized by the presence of an inflammatory component such as wound healing [18]. In addition, several clinical trials show their potential for storage for longer periods, easy collection, and avoidance of the side effects that could be associated with adult stem cells [19].

One of the most important components of MSC expansion is choosing a supplement in the culture medium that allows rapid cell propagation without affecting its characteristics [20]. In fact, while fetal bovine serum (FBS) could be associated with the risk of transmitting unknown animal diseases and inducing immune reactions versus animal antigens, human platelet lysate (hPL) has been demonstrated to be an animal serum-free substitute to FBS as a safer source of growth factors, allowing a better cell proliferation in vitro and showing promising results in clinical regenerative medicine applications [20,21].

This work demonstrates for the first time that the encapsulation of hUC-MSCs in gelatin–carrageenan hydrogels could represent a promising strategy for cell delivery systems in soft tissue regeneration. In particular, the developed hydrogel scaffolds present good swellability, structural integrity, and mechanical stability, with high compliance and viscoelasticity resembling the properties of soft natural tissues, as well as excellent strain reversibility under cyclic compression. Furthermore, they proved to be bioresorbable and biocompatible and to support the growth of hUC-MSCs with good viability and an increasing proliferation trend.

## 2. Results and Discussion

### 2.1. Physicochemical Properties of the Hydrogels

**Scheme 1:**
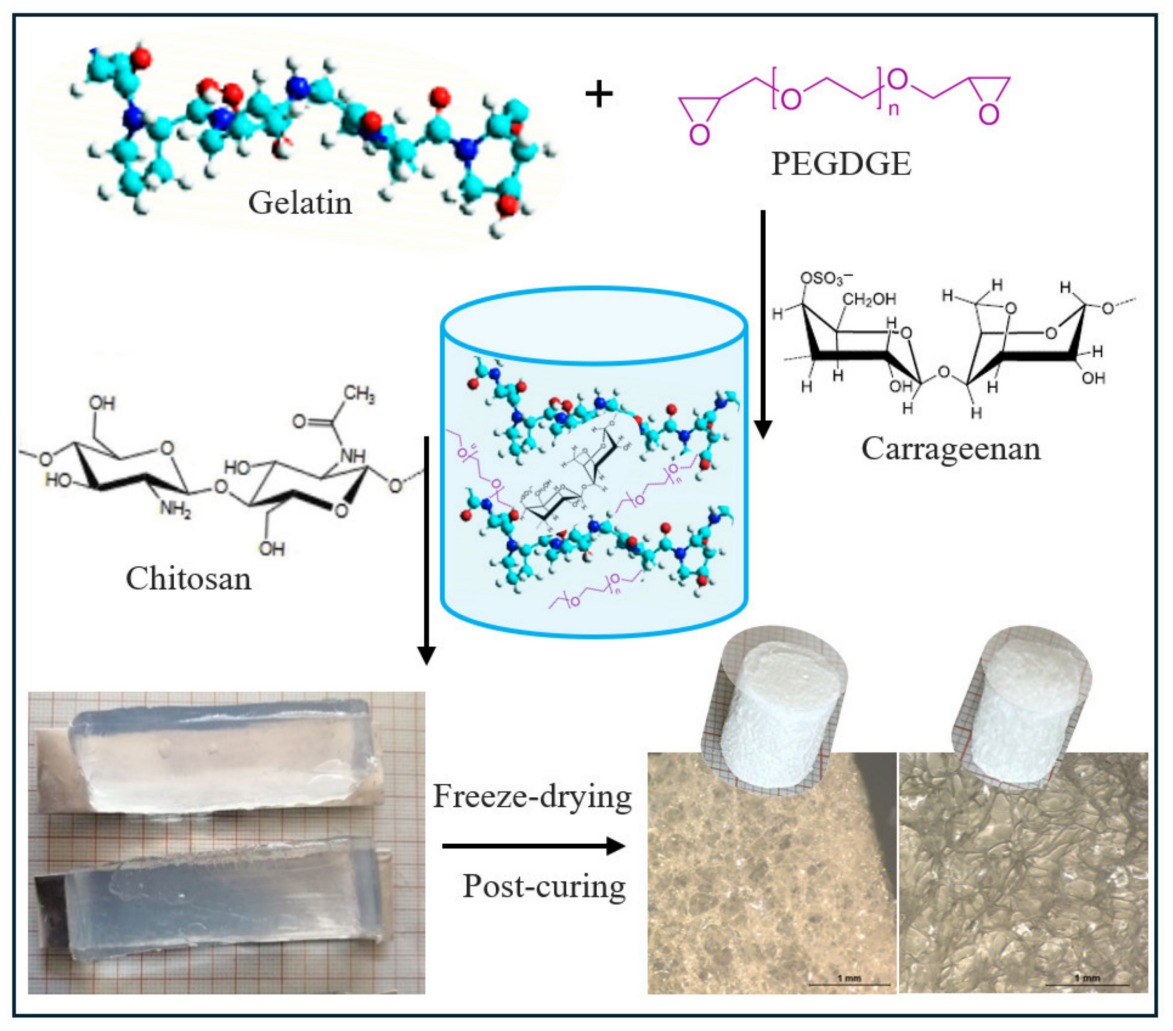
Diagram showing the hydrogels synthesis process.

To create structurally stable hydrogels in physiological settings, a variety of crosslinking techniques have been used. However, reports of a simple, easy-to-use, and green crosslinking technique are quite rare. Here, we propose a simple yet versatile synthesis strategy to develop stable hybrid hydrogels based on gelatin and supplemented with other natural components, in particular carrageenan with or without chitosan (G-PEG-CARR and G-PEG-CARR-CH, respectively) (Scheme 1). This strategy was previously optimized on gelatin–chitosan and gelatin–dextran derivatives [22,23], and is here applied to the new carrageenan-enriched formulations. To provide them with mechanical solidity and stability at body temperature, the hydrogels are covalently crosslinked with functionalized PEG (PEGDGE). Compared to conventional low molecular weight crosslinkers, PEGDGE enables the exploitation of epoxy groups’ ability to react with a variety of available functional groups, including secondary amino groups, carboxylic groups, and sulfate ester groups of carrageenan, in both acidic and basic conditions, in addition to the preferred primary amino groups of gelatin and chitosan. The combination of polypeptide and polysaccharide components in this multifunctional complex system can produce a biomimetic macromolecular network showing improved interactions with cells and natural molecules, also thanks to electrostatic interactions with negatively charged sulfate groups of carrageenan and positively charged groups in chitosan. In addition, in G-PEG-CARR-CH, both types of charge are present, and this may play a synergistic effect on the physicochemical properties of the material, but also affect the reactivity and solubility of the components. Nevertheless, a stable hybrid hydrogel network is achieved for both G-PEG-CARR and G-PEG-CARR-CH, with the final compositions detailed in Table 1.

**Figure 1:**
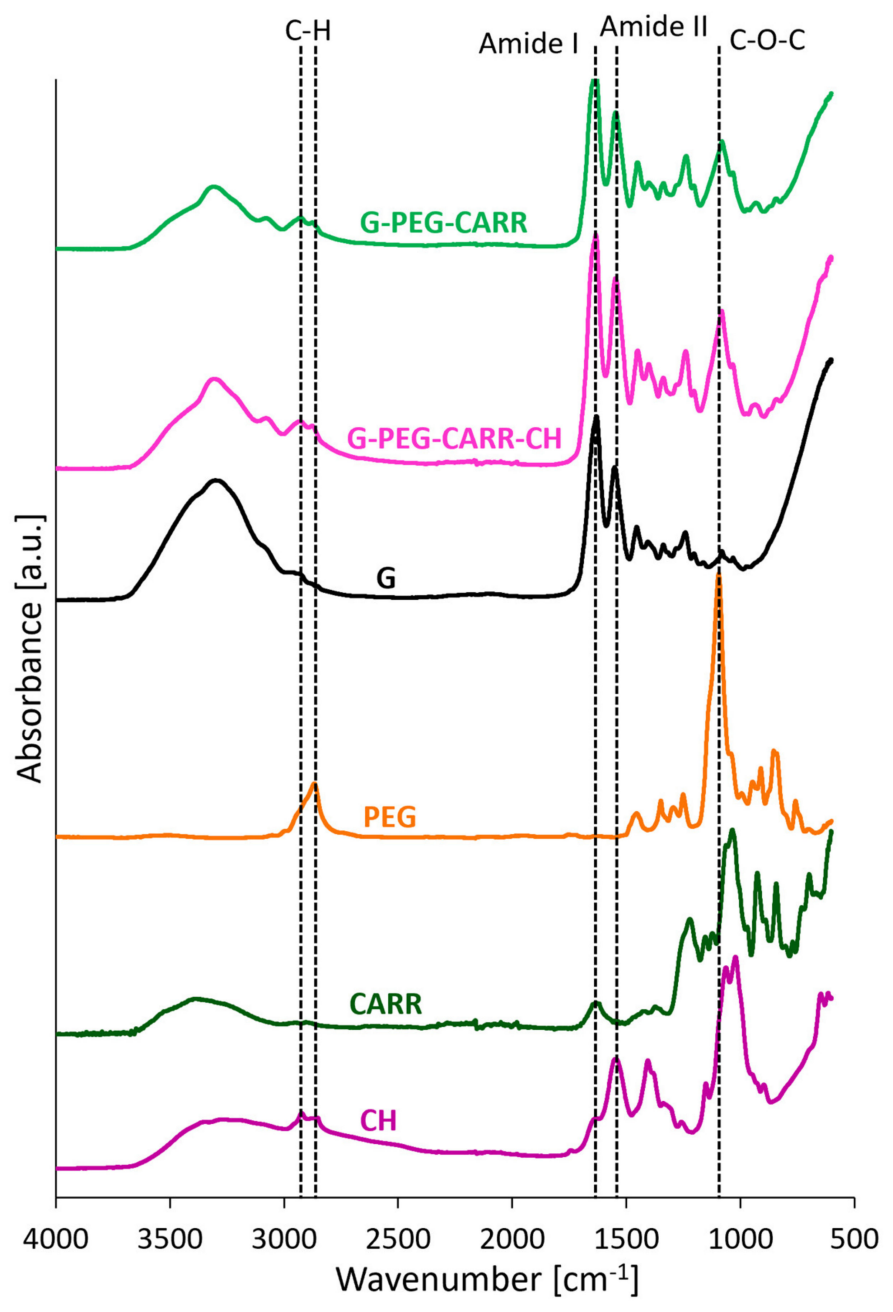
FTIR spectra of G-PEG-CARR and G-PEG-CARR-CH hydrogels and of their pure components.

FTIR analysis was done on the hydrogels after washing out the soluble and eventually unreacted components to confirm the chemical structure. Figure 1 presents the FTIR spectra of pure components and hybrid hydrogels. As shown in the spectra, all characteristic peaks of pristine components were observed in the two hydrogels; in particular, the absorption peaks around 2925–2875, 1633, 1548, and 1081 cm^−1^, correspondingly ascribed to C-H aliphatic stretching vibration, amide I (C-O stretching vibrations), amide II (-NH bending vibrations and C-N stretching vibrations), and C-O stretching vibrations (ether bond C-O-C) appeared in both hydrogel spectra. One notes a slight shift in the amide II band of crosslinked gelatin-based hydrogels compared with that of pure gelatin (1552 cm^−1^), indicating that the amide groups are involved in the crosslinking process [22]. It is worth noting that the representative intense bands exhibited around the 1236 cm^−1^ (S=O stretch of sulfate esters salts) and 1010–1080 cm^−1^ region (ascribed to glycosidic linkage and C-O stretch of cyclic ethers) confirmed the presence of CARR in both final hydrogels. The representative intense peak exhibited around 1081 cm^−1^, assigned to C–O stretching (ether bond C–O–C), also confirmed the presence of PEG in the final hydrogels. Overall, it is clear that hydrogel spectra have the characteristic peaks of gelatin and PEG with some shifts due to the interaction between PEG and gelatin in presence of carrageenan and chitosan.

Comparatively, the spectra of G-PEG-CARR and G-PEG-CARR-CH showed very similar bands and peaks. This is likely caused by the overlap of bands of these complex systems, due to the similarity in the IR-detectable groups of the macromolecular components, and by the low concentration of polysaccharides in the final hydrogel formulations, which may exceed the detection limit of spectroscopic analysis.

**Figure 2:**
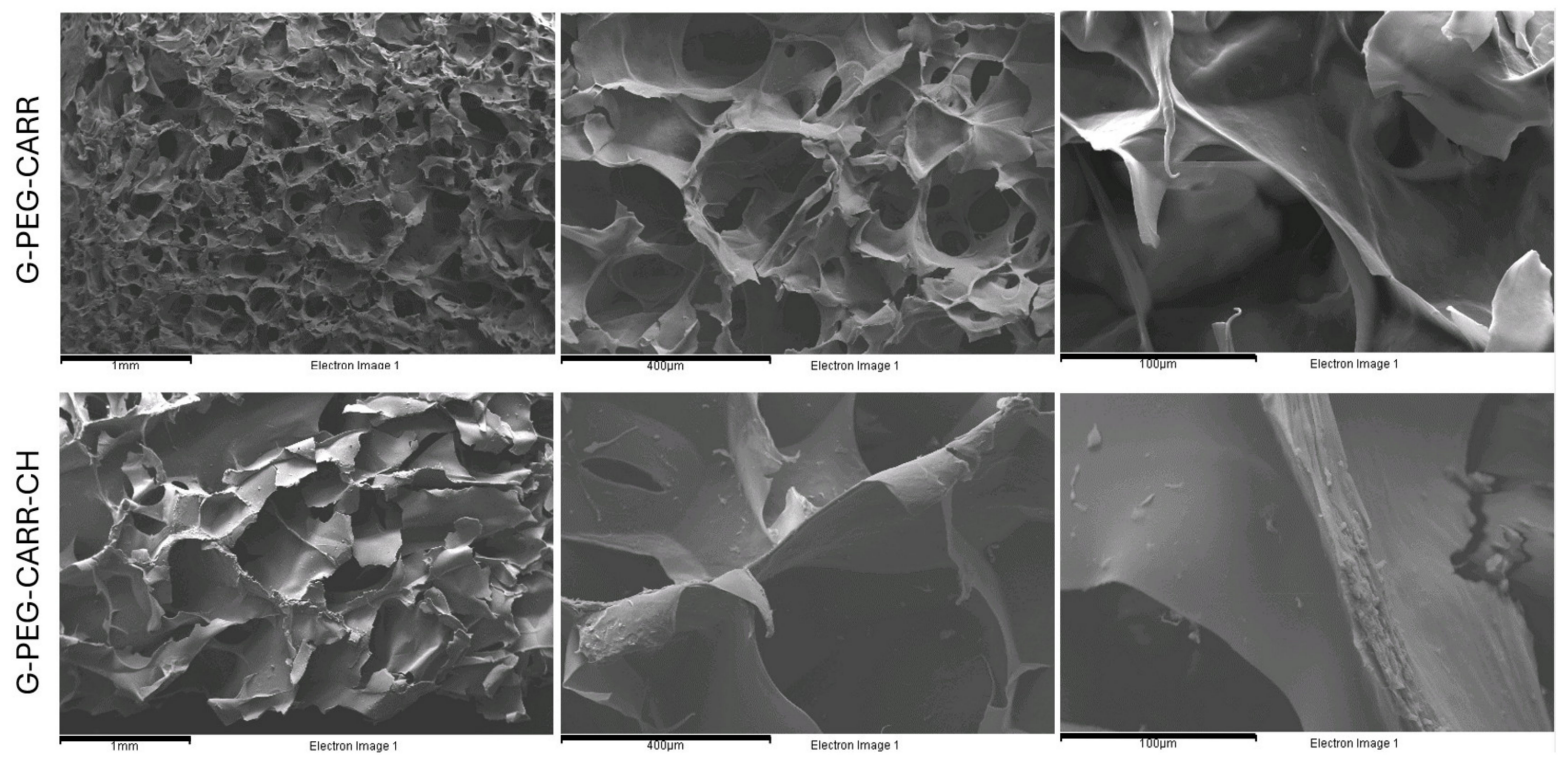
SEM images displaying the cross-section of dry hydrogels G-PEG-CARR and G-PEG-CARR-CH at increasing magnification. Scale bars from left to right: 1 mm; 400 µm; 100 µm.

As reported in Table 1, the two materials present similar apparent density (around 0.075–0.080 g/cm^3^) and porosity (about 80%) in dry conditions. Furthermore, they are both provided with a well-interconnected open porous structure, but G-PEG-CARR pores tend to have smaller and more homogeneous dimensions (rarely exceeding 200 µm), while G-PEG-CARR-CH presents a more heterogeneous network of channels, including pores between about 100 and 800 µm, as well as thicker microporous polymeric lamellae (up to 20 µm wide). These morphological differences can be appreciated in Figure 2, portraying the cross-sections of the two hydrogels as observed by means of a SEM at increasing magnification.

**Figure 3:**
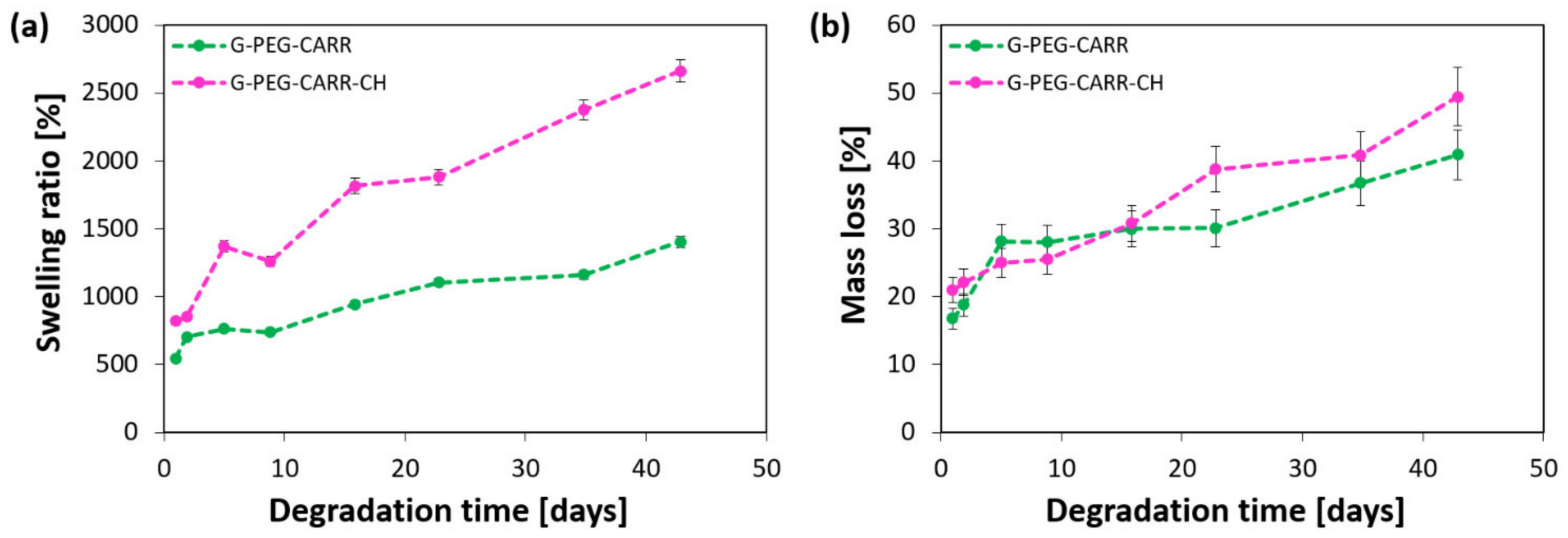
Swelling ratio (**a**) and mass loss (**b**) of G-PEG-CARR and G-PEG-CARR-CH hydrogels at various time points during prolonged immersion in distilled water at 37 °C.

In addition, Table 1 reports the values of the hydrogel swelling ratio in wet conditions before performing mechanical tests (i.e., after 24 h in distilled water). G-PEG-CARR-CH, with a swelling ratio of about 830%, absorbs significantly higher amounts of water with respect to G-PEG-CARR (550% ca.), which may be reasonably ascribed to a lower degree of crosslinking density. This difference further grows upon prolonged immersion in water, as displayed in Figure 3a; for example, after 43 days in water at body temperature, the swelling ratio reaches about 1400% for G-PEG-CARR and 2700% for G-PEG-CARR-CH. At the same time, G-PEG-CARR-CH exhibits higher mass loss values (Figure 3b), losing up to 50% of its dry weight at the end of the degradation experiments, compared to about 40% of mass loss by G-PEG-CARR. Importantly, the mass loss due to hydrolysis occurs gradually, so that macroscopic debris can be found only after 43 days in water at 37 °C, signing the conclusion of the experiments. Until then, the specimens maintain their integrity, also enabling mechanical testing after long degradation times (see Section 2.2).

### 2.2. Mechanical Properties of the Hydrogels

The mechanical behavior of the hydrogels was first investigated by uniaxial tensile and compression tests, obtaining information about their stiffness and strength under such loading conditions, as well as their ultimate tensile strain. Cyclic compression experiments were then carried out in order to explore the ability of the materials to recover strain and their dissipation energy under repeated loading–unloading. These tests served also as preconditioning of the samples, to stabilize their mechanical behavior before studying their stress relaxation response [24]. All these analyses were conducted after immersion of the specimens in distilled water for 24 h. Finally, the evolution of compression properties was studied during hydrolytic degradation at body temperature (37 °C).

**Figure 4:**
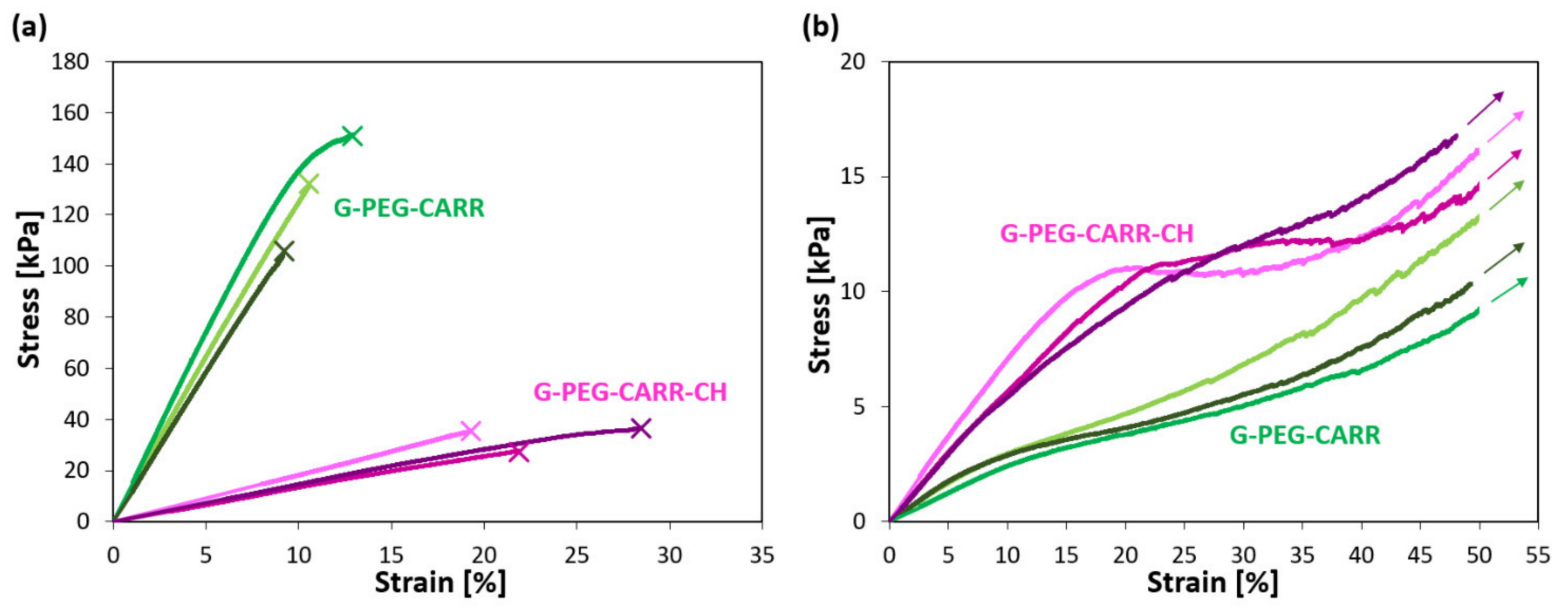
Stress–strain curves of G-PEG-CARR and G-PEG-CARR-CH wet hydrogels under (**a**) tensile and (**b**) compressive conditions.

Examples of stress–strain curves recorded during tensile tests are illustrated in Figure 4a and the derived mechanical properties are reported in Table 2. The curves exhibit a well-developed linear portion, where the elastic modulus is measured, and after which they show only a slight decrease in slope, usually at strain values well above 5% for G-PEG-CARR and 10% for G-PEG-CARR-CH. In the end, a crack propagates in the porous structure of the hydrogel until crossing the whole transversal section and breaking the specimen; ultimate tensile stress and strain values are considered only for specimens that fractured far enough from the clamping area. The obtained results show that G-PEG-CARR is significantly stiffer and stronger under tensile conditions with respect to G-PEG-CARR-CH (average tensile modulus: 1400 kPa vs. 140 kPa; average stress at break: 130 kPa vs. 36 kPa), while reaching lower average values of strain at break compared to the softer hydrogel (11% vs. 26%). Despite these differences, both hydrogels should be considered for regenerative applications involving soft biological tissues, given their high compliance.

Regarding compression tests, the obtained stress–strain curves (Figure 4b) show three different regions, as typical of cellular materials [25]: (i) a linear elastic region, once again reaching strain values of at least 5% for G-PEG-CARR and 10% for G-PEG-CARR-CH, and allowing for compressive modulus measurement; (ii) a transition region with lower stiffness, due to progressive collapse of the walls and lamellae forming the hydrogel microstructure, and beginning with a knee in the curve that appears smoother for G-PEG-CARR and more pronounced for G-PEG-CARR-CH; (iii) a densification region with strain-hardening due to the pore walls finally coming in contact with each other, typically for strains higher than 30–40%. Compressive data are recorded until strain values of about 50%, and compressive strength is here evaluated as the stress at 50% strain, that is taken as reference to compare the performance of the two materials. With respect to their tensile behavior, the two materials appear more compliant, as the walls and lamellae forming their cellular structure can easily bend; interestingly, this increased compliance under compression can be found also in some soft tissues such as skin and cartilage [26,27,28]. In addition, G-PEG-CARR exhibits lower values of compressive properties with respect to G-PEG-CARR-CH (average compressive modulus: 31 kPa vs. 63 kPa; average stress at 50% strain: 11 kPa vs. 16 kPa), a possible reason being the higher swelling of the latter combined with a more difficult release of water from denser pore walls during compression [29].

**Figure 5:**
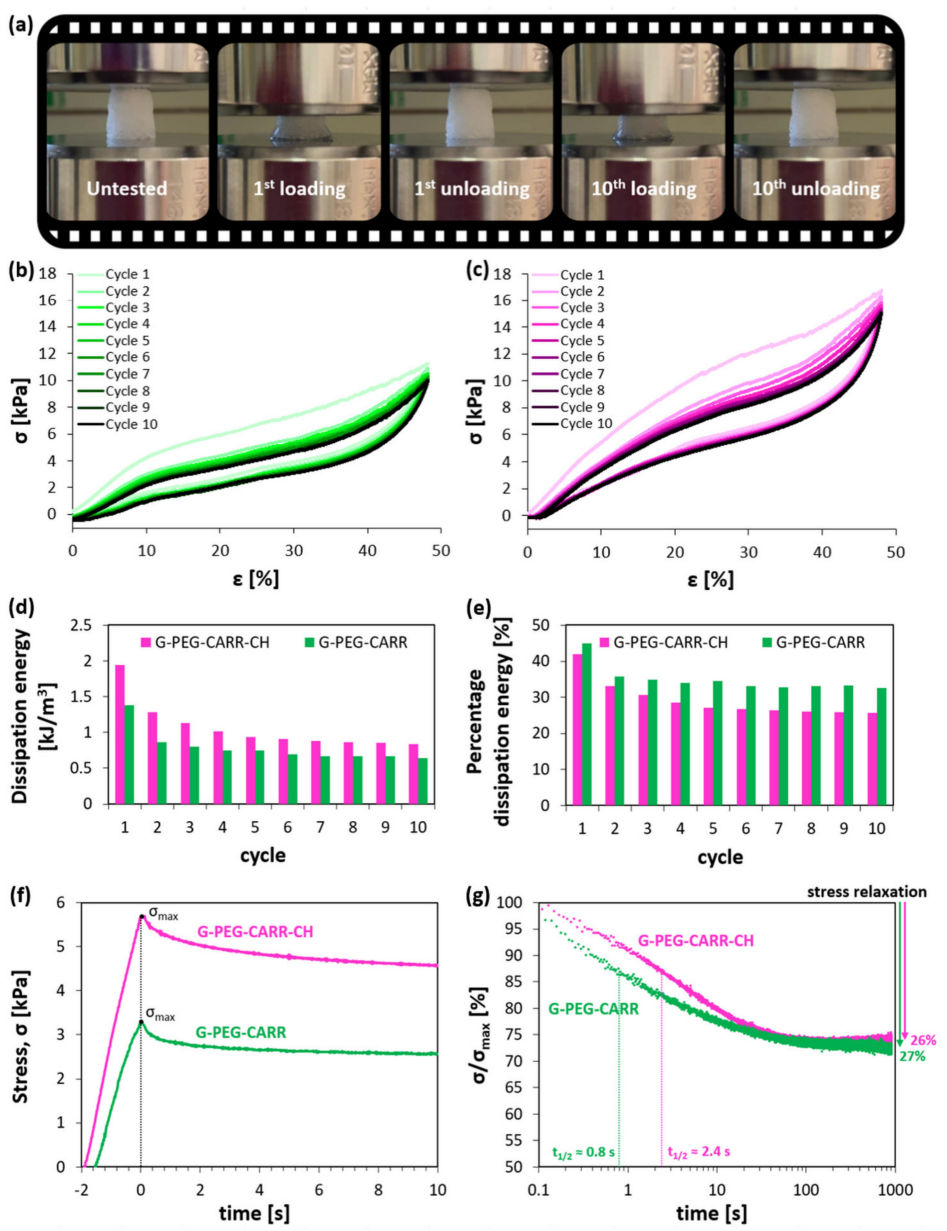
(**a**) G-PEG-CARR specimen before and after the 1st and 10th compression cycles up to 50% strain; (**b**) stress vs. strain curves of (**b**) G-PEG-CARR and (**c**) G-PEG-CARR-CH hydrogels during cyclic compression tests (preconditioning); (**d**) dissipation energy and (**e**) percentage dissipation energy associated with each cycle; (**f**) stress and (**g**) normalized stress plotted against time during stress relaxation tests on preconditioned specimens.

Further compression tests were conducted to study the two hydrogel systems also in terms of viscoelasticity, since the scaffold viscoelastic behavior is known to play an important role in the mimicry of biological tissues and in the stimulation of cell responses, being especially promoted by faster stress relaxation [30]. The purpose of doing cyclic compression tests is to show whether the hybrid hydrogels exhibit natural tissue-like preconditioning, characteristic hysteresis, nonlinear elasticity, and energy dissipation. Figure 5 reports the results of cyclic compression and stress relaxation experiments, performed as 10 loading–unloading cycles at 50% strain immediately followed by stress relaxation at 15% for 15 min. After 24 h immersion in water, both hydrogels are able to repeatedly recover the applied large strains, accumulating negligible residual strains within 1% after the first cycle and 2% after 10 cycles (see Figure 4a).

Stress–strain plots of the 10 cycles for G-PEG-CARR (Figure 5b) and G-PEG-CARR-CH (Figure 5c) show distinct loading and unloading curves, forming hysteresis loops typical of viscoelastic materials, and the occurrence of progressive stress softening (Mullins effect) as the number of cycles increases; the main changes occur during the first loading–unloading cycle, then the curves tend to overlap and mechanical stability is achieved after about five cycles. The Mullins effect has been often observed in soft materials as filled elastomers and gels [31,32], and previously encountered also in freeze-dried gelatin-based hydrogels by Dey et al. [33]; in particular, in the case of hydrogels, the stress softening is due to irreversible internal microfractures. This phenomenon does not compromise the structural integrity nor the strain reversibility of G-PEG-CARR and G-PEG-CARR-CH, but can significantly affect their dissipation energy and stress relaxation. Indeed, the hysteresis loop area progressively decreases with the number of cycles, indicating a reduction in dissipation energy; during the last cycles, a stable value of dissipation is recorded, and in the end the stress relaxation is evaluated at 15% strain, within typical strain levels exerted by cells in 3D cell culture [34]. The need for preconditioning the sample before achieving stable mechanical properties is well known also in biological material testing [24,35], as further manifestation of the similarity between hydrogels and the extracellular matrix of living tissues.

More in detail, G-PEG-CARR dissipates slightly less energy than G-PEG-CARR-CH in absolute values (Figure 5d), but more in terms of percentage dissipation energy, calculated with respect to the total area under loading stress–strain curves (about 33% vs. 26% during the last cycles, Figure 5e). It also exhibits a faster percentage stress relaxation, as observed by comparing their reduced relaxation function, according to Pinto and Fung’s definition [36]: first, the time is set to zero as soon as the desired strain (15%) is applied, and the corresponding peak is identified as $\sigma_{max}$, as shown in Figure 5f; then, the reduced relaxation function is obtained by normalizing the stress values to $\sigma_{max}$ and plotting them against time, as reported in Figure 5g. Notably, the two materials in this study present similar values of percentage stress relaxation (26–27%), but G-PEG-CARR relaxes three times faster than G-PEG-CARR-CH (half stress relaxation time, $t_{1/2}$: 0.8 s vs. 2.4 s). Together with its higher percentage dissipation energy, this is a sign of the more pronounced viscoelastic nature of G-PEG-CARR, which may particularly hint at its suitability for soft tissue engineering, especially in the case of tissues bearing repeated loads such as skin, muscles, or cartilage. Anyway, both the covalent-crosslinked materials here proposed exhibit considerably fast stress relaxation compared to many other hydrogels reported in the literature, especially compared to other systems based on covalent bonds; this is likely due to a combination of slower viscoelastic response typical of covalent-crosslinked networks, involving the movement of entangled macromolecules or loose ends of polymer chains, with faster poroelastic relaxation caused by the movement of water [29,37,38].

**Figure 6:**
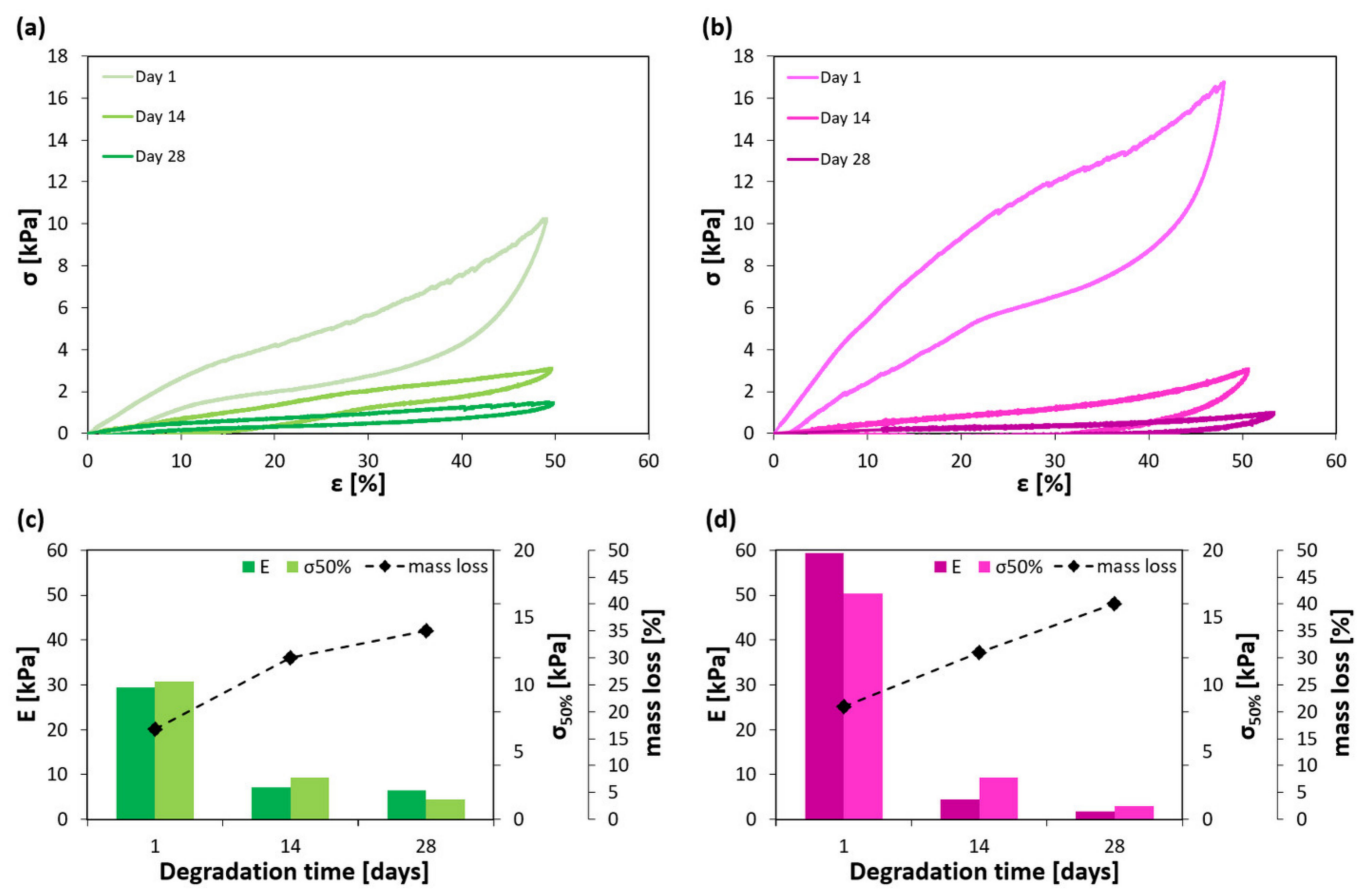
Stress–strain compressive curves of (**a**) G-PEG-CARR and (**b**) G-PEG-CARR-CH hydrogels immersed in water at 37 °C for 1, 14, or 28 days, compressed until 50% strain and unloaded; compressive modulus, stress at 50% strain and mass loss of (**c**) G-PEG-CARR and (**d**) G-PEG-CARR-CH hydrogels for the different degradation times.

Finally, it was verified that, despite their degradation, the hydrogels preserve structural integrity and can sustain mechanical stresses after several weeks. In fact, it is fundamental that the hydrogels maintain the overall shape and volume during the time necessary for new tissue formation. The evolution of hydrogel mechanical properties during hydrolytic degradation at 37 °C is summarized in Figure 6. One loading–unloading curve is reported for each time point (1, 14, and 28 days) for both G-PEG-CARR (Figure 6a) and G-PEG-CARR-CH (Figure 6b), subjected to compression up to 50% strain. The corresponding values of compression modulus, stress at 50% strain, and mass loss are illustrated in Figure 6c (G-PEG-CARR) and Figure 6d (G-PEG-CARR-CH). As expected from the mass loss profiles, a progressive reduction in mechanical performance is found for both materials as degradation occurs. While the values of compression modulus, stress at 50% strain, and hysteresis loop area decrease, the residual deformation at unloading increases, indicating only partial strain recovery capability in the degraded hydrogels. All these changes tend to be faster for G-PEG-CARR-CH, consistently with its overall higher swelling ratio and mass loss trends. Altogether, both materials should be dedicated to the reconstruction of fast-regenerating soft tissues, such as skin, rather than the treatment of tissues having long healing times. The ability of the two hydrogels to significantly support hUC-MSCs’ viability and proliferation within a few weeks of cell culture is documented in Section 2.3, describing the results of in vitro experiments.

### 2.3. Results of In Vitro Studies on Hydrogel Scaffolds

#### 2.3.1. hUC-MSCs Maintained Viability into the G-PEG-CARR and G-PEG-CARR-CH Scaffolds

**Figure 7:**
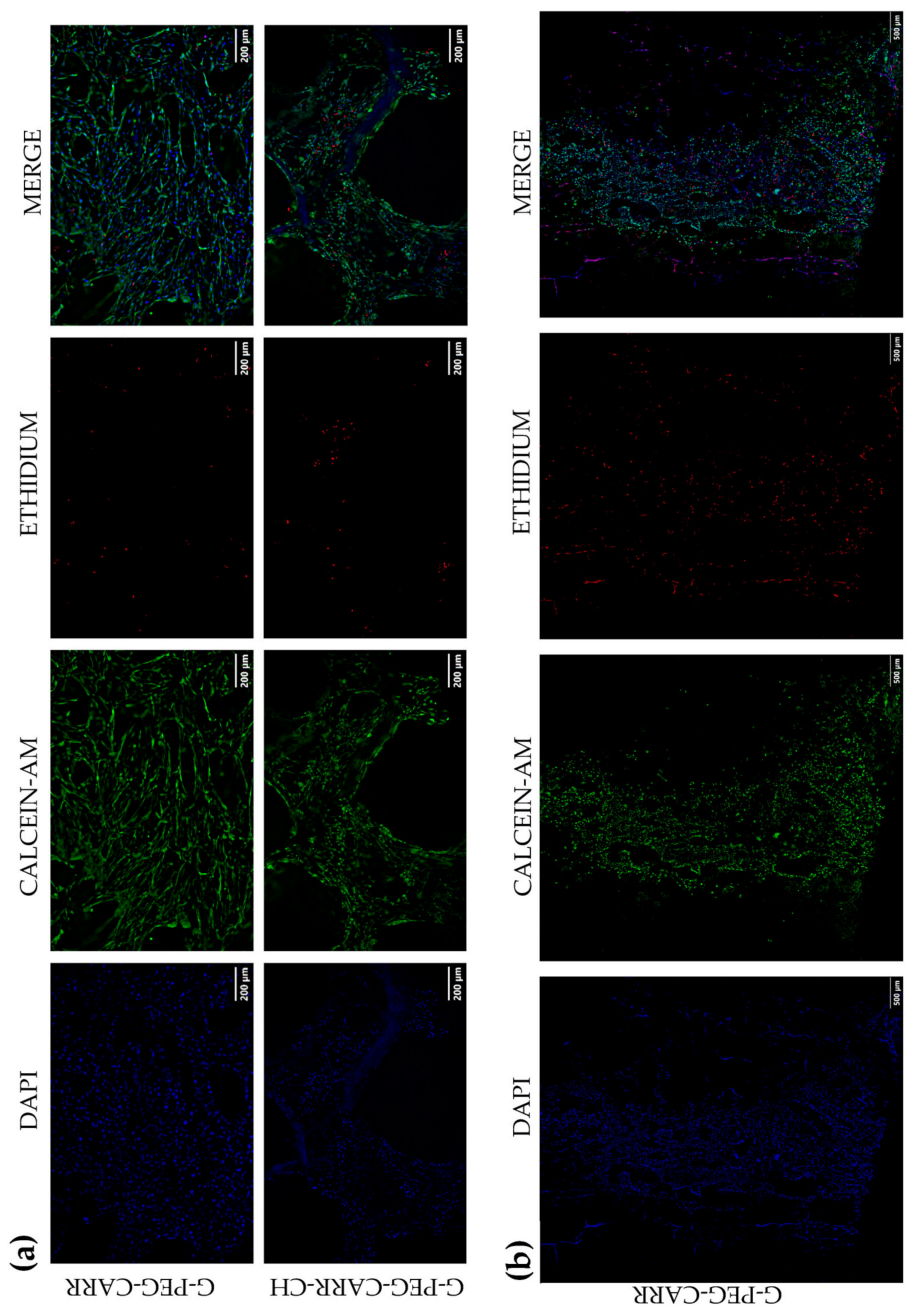
Panel of images illustrating the viability of hUC-MSCs seeded into G-PEG-CARR and G-PEG-CARR-CH scaffolds after 3 days of culture in complete medium + 5% hPL measured by Live/Dead assay. All images were obtained using the Olympus IX83 fluorescence microscope. Staining of nuclei (DAPI), viable cells (Calcein-AM), dead cells (Ethidium), and merge of all channels are reported. (**a**) Images obtained with 20X zoom representing the viability of hUC-MSCs into the two scaffolds. Scale bar: 200 µm. (**b**) Images obtained with 4X zoom representing almost the totality of a G-PEG-CARR sample. Scale bar: 500 µm.

**Figure 8:**
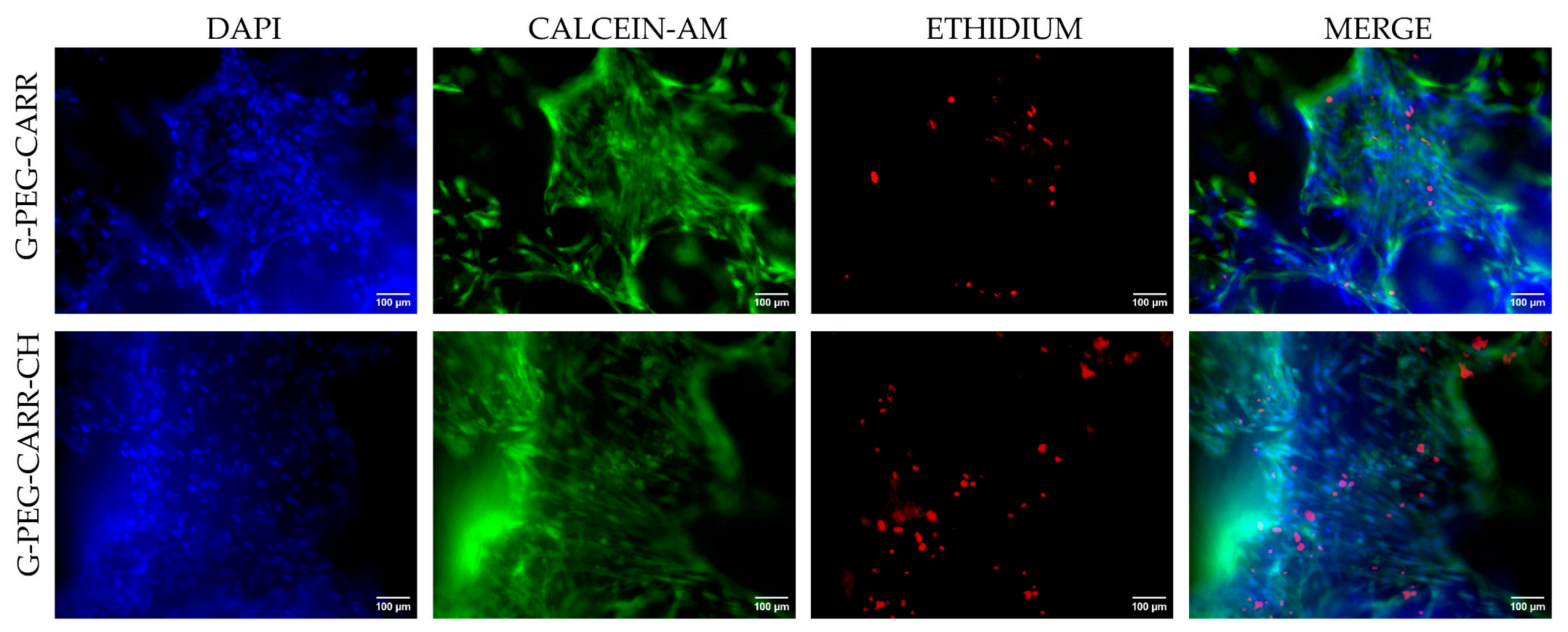
Panel of images illustrating the viability of hUC-MSCs seeded into G-PEG-CARR and G-PEG-CARR-CH scaffolds after 21 days of culture in complete medium + 5% hPL, measured by Live/Dead assay. All images were obtained using the Olympus IX70 fluorescence microscope with a 10X zoom. Scale bar: 100 µm. Staining of nuclei (DAPI), viable cells (Calcein-AM), dead cells (Ethidium), and merge of all channels are reported.

At day 3 and day 21 of 3D culture, cell viability was evaluated using a Live/Dead assay, in order to appreciate the cell colonization and distribution throughout the structure of both hydrogels (Figure 7a,b and Figure 8, respectively). DAPI staining of nuclei confirm the presence of cells in both G-PEG-CARR and G-PEG-CARR-CH scaffolds. Calcein-AM and EthD-1 staining allow to appreciate the viable cells in green and the dead cells in red. The Live/Dead assay shows that cells are viable not only after 3 days but even after 21 days of culture (Figure 8) and maintained their fibroblastic-like morphology. hUC-MSCs are equally distributed into G-PEG-CARR and G-PEG-CARR-CH hydrogels regardless of the time point, both in the center and along the edges of the scaffolds, showing great affinity for these novel carrageenan-based formulations. In detail, hUC-MSCs are distributed more homogenously on the entire structure of G-PEG-CARR scaffolds, while hUC-MSCs are more concentrated on the meshes of G-PEG-CARR-CH with lesser permanence into the porosities (Figure 7a). In fact, the scaffolds show different porosity, and this could influence the colonization and permanence of cells within the material: together with diffused microporosities, G-PEG-CARR tends to have smaller macropores (typically within 200 µm) with a homogeneous distribution, while G-PEG-CARR-CH presents a more heterogeneous network of channels, with the presence of larger pores (between about 100 µm and 800 µm). The porosity features of G-PEG-CARR scaffolds allow a more uniform distribution and attachment of the hUC-MSCs on both the meshes and the porosities of the scaffolds.

It is noteworthy that macropores around at least 100 μm, depending on the type of cells used, are necessary for the homogenous distribution of cells throughout the scaffold and to allow effective in vivo tissue regeneration [39]. On the other hand, micropores ranging around 1–10 μm, better promote protein adsorption, cell adhesion, and cell proliferation on the scaffolds, as well as angiogenesis [40,41]. Scaffolds containing both macropores and micropores exploit the advantages of both pore sizes and have excellent biocompatibility properties [41].

In Figure 7b, a G-PEG-CARR scaffold can be appreciated in its almost totality after 3 days of cell culture, observing both its 3D porous structure and its colonization by cells. The cells tend to be concentrated at the seeding point on the scaffold and gradually spread through the porous structure, thanks to the high pore interconnectivity of G-PEG-CARR.

**Figure 9:**
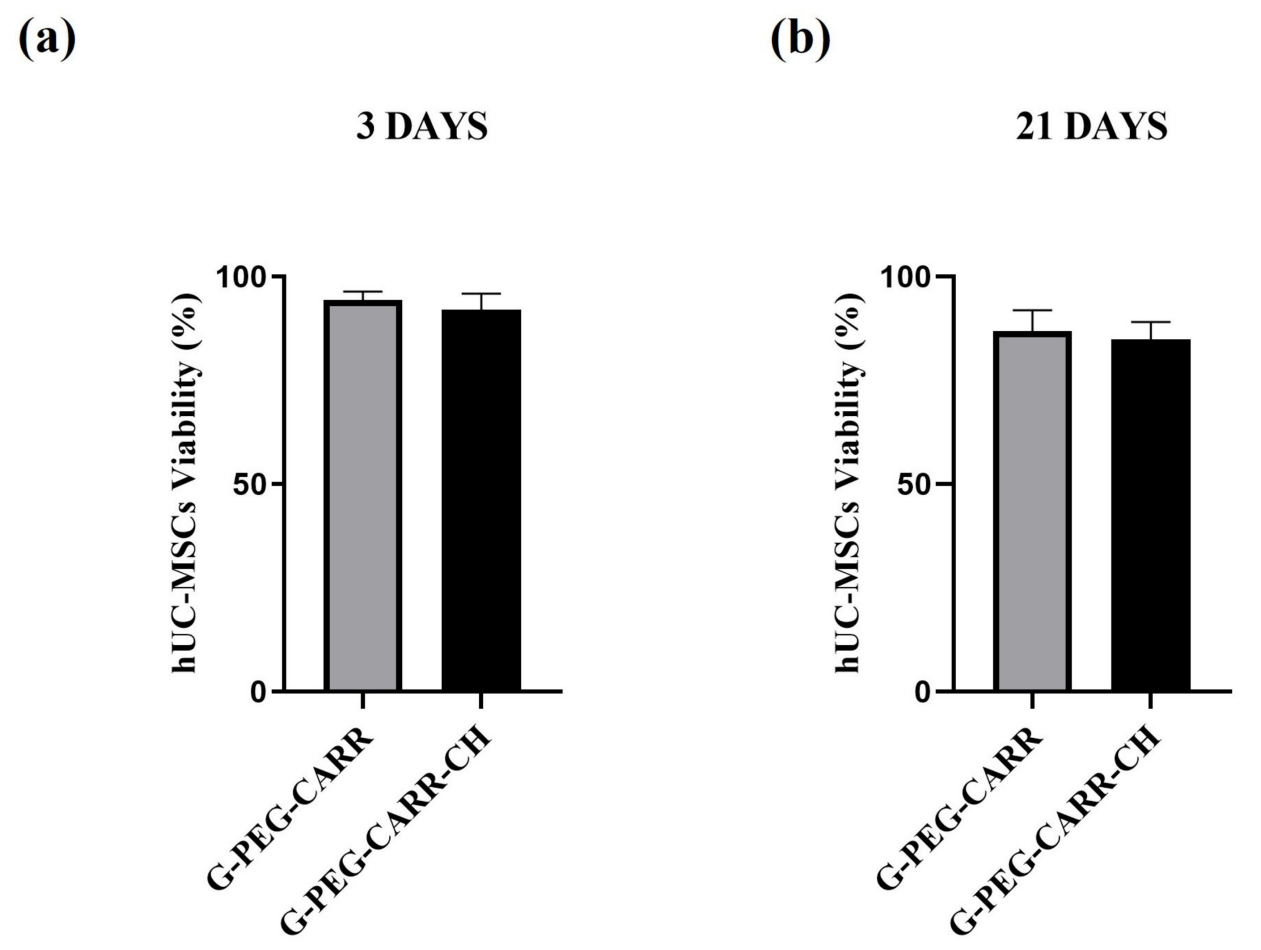
Viability of hUC-MSCs, expressed as percentage, cultured in G-PEG-CARR and G-PEG-CARR-CH hydrogels in complete medium + 5% hPL after (**a**) 3 days and (**b**) 21 days and examined by Live/Dead images using ImageJ software (Version 1.51). Data are expressed as mean ± standard deviation and do not show significant differences.

No significant differences in the viability of hUC-MSCs, expressed as percentage, were noted in both the two types of scaffolds and time points (G-PEG-CARR, 3 days, 94.26% ± 2.11; G-PEG-CARR-CH, 3 days 91.97% ± 3.95; G-PEG-CARR, 21 days, 86.77% ± 5.15; G-PEG-CARR-CH, 21 days, 84.90% ± 4.15) (Figure 9).

#### 2.3.2. hUC-MSCs Proliferate with an Increasing Trend into the Scaffolds

**Figure 10:**
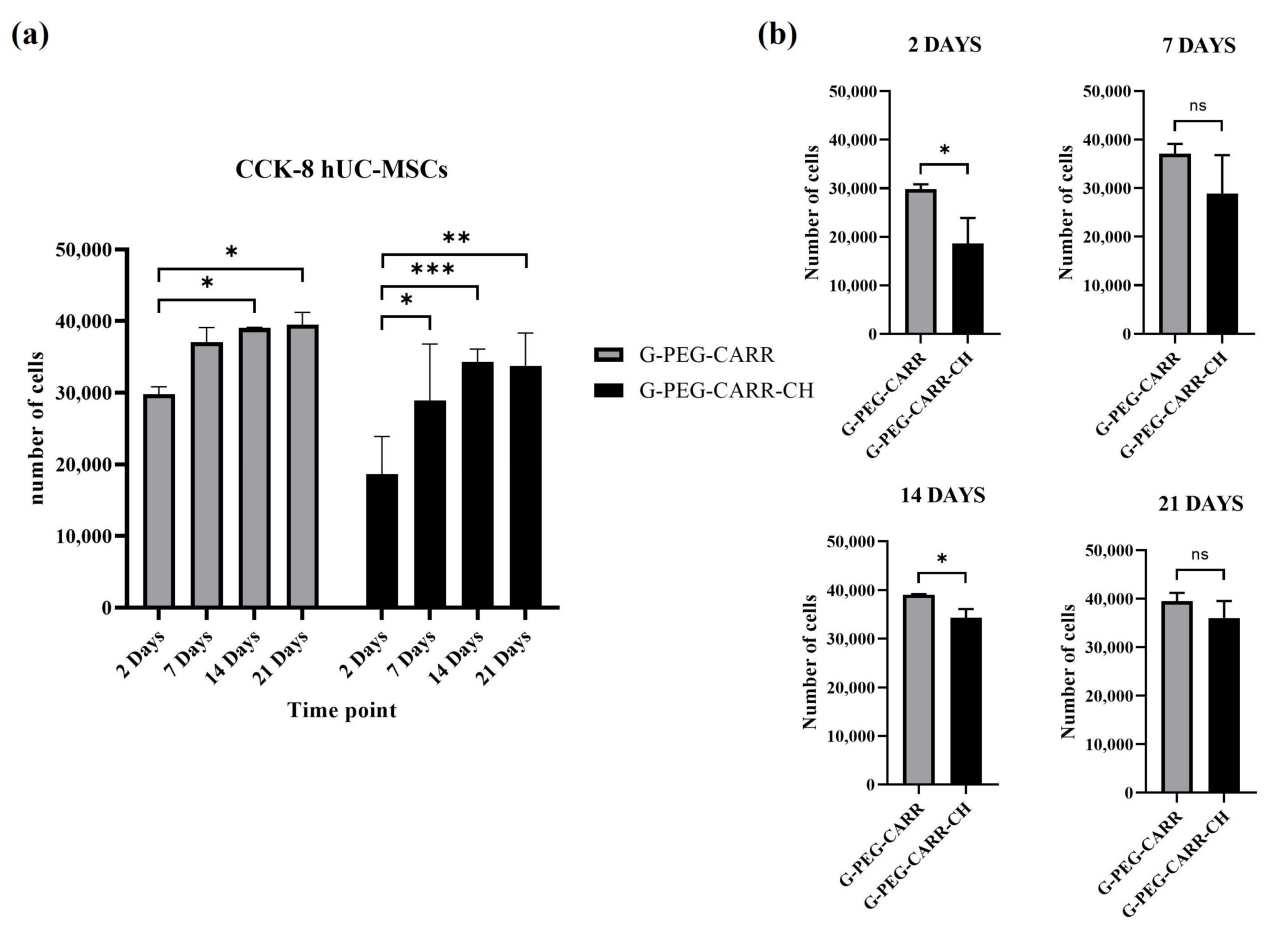
Proliferation of hUC-MSCs, expressed as absolute number of cells, cultured in G-PEG-CARR and G-PEG-CARR-CH hydrogels, measured by CCK-8 assay in complete medium + 5% hPL at different time points (2, 7, 14, 21 days). (**a**) Total graph representing hUC-MSCs’ proliferation in G-PEG-CARR and G-PEG-CARR-CH scaffolds at different time points. (**b**) Comparison graphs of cells behavior in both scaffolds at the same time point. Statistical analysis was performed using (**a**) two-way ANOVA with Tukey’s post hoc test and (**b**) unpaired *t*-test. Data are expressed as mean ± standard deviation and show significant differences (* *p ≤* 0.05, ** *p ≤* 0.01, *** *p ≤* 0.001, ns: no statistical significance).

Cell proliferation was measured by reading absorbance at 450 nm at different time points after cell seeding into scaffolds and calculating the corresponding cell number using a calibration curve, following manufacturer instructions. The results obtained show that hUC-MSCs proliferate along time with an increasing trend into both type of scaffolds, until finally reaching a plateau phase at 21 days of 3D culture (Figure 10a). In particular, considering the proliferation of cells within the G-PEG-CARR scaffold, it is statistically significant when comparing the number of cells at day 2 versus days 14 and 21 of culture (G-PEG-CARR day 2 vs. G-PEG-CARR day 14, * *p* = 0.0471; G-PEG-CARR day 2 vs. G-PEG-CARR day 21, * *p* = 0.0363) (Figure 10a). Instead, considering the cells proliferating into the G-PEG-CARR-CH scaffold, a statistically significant result is obtained when comparing the number of cells at day 2 with respect to all other time points (G-PEG-CARR-CH day 2 vs. G-PEG-CARR-CH day 7, * *p* = 0.0250; G-PEG-CH-CARR day 2 vs. G-PEG-CARR-CH day 14, *** *p* = 0.0008; G-PEG-CARR-CH day 2 vs. G-PEG-CARR-CH day 21, ** *p* = 0.0012) (Figure 10a). Comparing the other time points with each other (day 7 vs. day 14 vs. day 21) there is no statistically significant difference. Absorbance values obtained at day 21 are comparable to those obtained at day 14, indicating that cells have already reached the maximum degree of growth at the time points considered (Figure 10a). Even if the proliferation kinetic of the hUC-MSCs was different for the two scaffold types, the cells reached a similar absolute number around 4 × 10^4^ cells in both G-PEG-CARR and G-PEG-CARR-CH at the last time point investigated. In addition, a significantly higher number of cells is observed in G-PEG-CARR both at day 2 and day 14 (G-PEG-CARR day 2 vs. G-PEG-CARR-CH day 2, * *p* = 0.0225; G-PEG-CARR day 14 vs. G-PEG-CARR-CH day 14, * *p* = 0.0103) (Figure 10b).

Finally, all these results indicate that G-PEG-CARR appears to be superior in terms of the absolute cell number within the material, while G-PEG-CARR-CH shows a better increasing trend in terms of proliferation. This hypothesis could be related to the different ranges of porosity characterizing the two types of scaffolds: the smaller pores of G-PEG-CARR could favor a better colonization and permanence of cells after seeding; on the other hand, the wide pores of G-PEG-CARR-CH could lead to a greater release of cells after seeding (referred to the lower number of cells at 2 days of cell culturing), but at later time points a better proliferation trend in continuous growth could be observed on the latter. In addition, the cell release from G-PEG-CARR-CH scaffolds could be favored by the higher mass loss and swelling ratio values exhibited by G-PEG-CARR-CH with respect to G-PEG-CARR (Figure 3 and Figure 10a,b).

## 3. Conclusions

The development of novel hydrogel formulations or improvement of existing hydrogels is currently attracting a lot of attention from biomedical scientists, and has resulted in many commercial products. Combining the features and benefits of different components, hybrid hydrogels can act as scaffolds to promote the growth of cells and tissue regeneration, and additionally as pharmaceutical carriers, by accommodating oppositely charged drugs, macromolecules and proteins as targeting motifs.

In this paper, we successfully developed novel crosslinked hydrolytically degradable hybrid hydrogels based on gelatin and carrageenan with highly interconnected porosity and soft mechanical properties using a benign synthesis approach suitable for a broad range of biomedical applications.

The hydrogels demonstrated good structural and mechanical stability, and strain reversibility under repeated compressive cycles at 50% deformation. Moreover, the hysteresis loops, percentage dissipation energy and stress-relaxing behavior of both G-PEG-CARR and G-PEG-CARR-CH hybrid hydrogels indicated their natural tissue-like viscoelasticity, and their high compliance appeared particularly suitable for the treatment of soft tissues. The two materials also demonstrated bioresorbability and biocompatibility, supporting the adhesion and dissemination of hUC-MSCs with high viability and a trend toward increasing proliferation.

Finally, our findings showed that encapsulating hUC-MSCs in gelatin–carrageenan hydrogels could be a viable method for cell delivery systems in a range of biomedical applications, especially soft tissue regeneration. Potential applications in tissue engineering and/or controlled release of drugs/biomolecules deserve further studies on these innovative regenerative models, combining the biomimetic structure and mechanics of hybrid hydrogels with the high regenerative efficiency and therapeutic potential of hUC-MSCs.

## 4. Materials and Methods

### 4.1. Materials

Type A Gelatin, G (pharmaceutical grade, 280 bloom, viscosity 4.30 mP), was kindly supplied by Italgelatine (Cuneo, Italy). Poly(ethylene glycol) diglycidyl ether, PEGDGE (molecular weight 526 Da), chitosan, CH (molecular weight between 50,000 and 190,000 Da, degree of deacetylation 75–85%), and carrageenan, CARR (commercial carrageenan type I, containing predominantly κ-carrageenan with the least amount of λ-carrageenan, C1013 [42]), were purchased from Sigma Aldrich Co. (Milan, Italy). Acetic acid, ethylene diamine, and ethanol were obtained by Fluka (Milan, Italy). Dulbecco’s modified Eagle’s medium (DMEM) and Fetal Bovine Serum (FBS) were purchased from Sigma Aldrich Co. (Milan, Italy).

### 4.2. Preparation of Hydrogels

Gelatin-based scaffolds containing either carrageenan (G-PEG-CARR) or carrageenan and chitosan (G-PEG-CARR-CH) were realized. Highly porous hybrid hydrogels were prepared in aqueous solution using a simple freeze-drying technique followed by a post-curing reaction at 45 °C. The production process involved the use of functionalized poly(ethylene glycol) (PEGDGE) as crosslinker, through reactions of its epoxy groups with other functional groups such as the amino groups of gelatin and chitosan and the sulfate ester groups of carrageenan (Scheme 1). The final content of G, PEG, CARR, and CH in the dry samples is reported in Table 1.

#### 4.2.1. Preparation of the G-PEG-CARR Hydrogel

Gelatin (3 g) was dissolved in 30 mL of distilled water at 45 °C under mild magnetic stirring, followed by the slow addition of the crosslinker PEGDGE (0.7 g). A 1% solution of CARR (30.3 g) was added to the reaction mixture and, again under slow stirring at 45 °C, a few drops of ethylene diamine were added until pH between 7 and 7.5 was reached. The reaction mixture was maintained under stirring at 45 °C for 1 h and then poured into a glass plate for gelification at room temperature. The gel was incubated at −20 °C until complete freezing and then transferred to a lyophilizer (Edwards Modulyo freeze-dryer) for the sublimation of ice crystals. Finally, to further increase the degree of crosslinking/grafting reactions, the dry hydrogel was subjected to a post-reaction process at 45 °C for 2 h in an oven under vacuum. The dry hydrogel was cut into cylindrical pieces (diameter: 10 mm, height: 12 mm) as well as rectangular pieces (80 × 10 × 2 mm^3^) of each composition for mechanical tests. For in vitro analyses, additional rectangular pieces (8 × 4 × 5 mm^3^) were washed several times by immersion in distilled water at 37 °C for 24 h to eventually remove the unreacted reagents and dissolved components, then frozen and freeze-dried in the lyophilizer. The samples were packed into small polypropylene bags and sterilized by gamma irradiation (Co-60 gamma source) using a dose of 25 kGy according to UNI EN ISO 11137 (Sterilisation of Health Care Products) [43].

#### 4.2.2. Preparation of the G-PEG-CARR-CH Hydrogel

The second hydrogel was prepared as described in Section 4.2.1 with the only difference being that, after the addition of carrageenan, a 2% chitosan solution in acetic acid (16.5 g) was added.

### 4.3. Chemical Structure and Morphology Analysis

The chemical characterization of hydrogels was performed using Fourier transform infrared (FTIR) spectroscopy on dry hydrogels using a Thermo Scientific, Nicolet iS50 FTIR spectrophotometer equipped with a PIKE MIRacle attenuated total reflectance attachment. Spectra were recorded over a range of 400 to 4000 cm^−1^ at a resolution of 4 cm^−1^.

The morphology of the prepared materials was observed at the optical microscope (reflected light digital microscope, Leica DMS 300). Moreover, a Scanning Electron Microscope (SEM) (LEO EVO 40, Carl Zeiss AG, Oberkochen, Germany) was employed to better observe the morphology of the section of mechanically sawed specimens.

### 4.4. Evaluation of Apparent Density, Porosity, Swelling and Mass Loss

The apparent density and porosity of the materials in dry conditions were measured by ethanol displacement method [44]. Ethanol was selected as wetting agent because it can easily penetrate the hydrogel pores without inducing any shrinkage or swelling, and without dissolving the hydrogel at room temperature. A known volume of ethanol ($V_{1}$) and a known mass of dry hydrogel samples (W) were placed into a graded cylinder, obtaining the measure of their total volume ($V_{2}$); after withdrawing the hydrogel, the residual volume of ethanol ($V_{3}$) was also recorded. The volume difference ($V_{2}-V_{1}$) corresponds to the volume of the hydrogel skeleton, while ($V_{2}-V_{3}$) is the total hydrogel volume. The apparent density and the porosity of the hydrogels were measured according to the following equations.

(1) $$Apparent\;density=\frac{W}{V_{2}-V_{3}}$$

(2) $$Porosity=\frac{V_{1}-V_{3}}{V_{2}-V_{3}}$$

Finally, the percentage swelling ratio (%) and mass loss (%) of the materials were evaluated at various time points during prolonged immersion in distilled water at 37 °C, in order to study their swelling and hydrolytic degradation profiles. The samples were weighed in their initial dry conditions ($W_{i}$), in wet conditions ($W_{w}$), and after final vacuum drying at 45 °C for 2 h ($W_{f}$); in particular, samples were withdrawn from the aqueous solution after predetermined time intervals of 1, 2, 5, 9, 16, 23, 35, and 43 days. The swelling ratio and mass loss were calculated using the following equations.

(3) $$Swelling\;ratio\;\left[\%\right]=\frac{W_{w}-W_{f}}{W_{f}}\times 100$$

(4) $$Mass\;loss\;\left[\%\right]=\frac{W_{i}-W_{f}}{W_{i}}\times 100$$

The samples were triplicated, and the average values were exposed with standard deviation.

### 4.5. Mechanical Characterization

Mechanical tests were carried out at room temperature by means of an electromechanical dynamometer (Instron, Mod. 3366, Norwood, MA, USA) with a 50 N load cell. Specimens were subjected to uniaxial tension, compression, cyclic compression and stress relaxation tests after immersion in distilled water for 24 h; compressive properties were also evaluated after hydrolytic degradation in distilled water at 37 °C for 1, 14 and 28 days. The dimensions of the wet specimens were measured by means of the software ImageJ (National Institutes of Health, Bethesda, MD, USA) on images acquired with an optical microscope (Leica DMS 300) right before testing. Engineering stress (σ) was calculated by dividing the recorded force by the initial cross-sectional area. Engineering strain (ε) was calculated by dividing the crosshead displacement by the initial gauge length (for tensile tests) or by the initial specimen height (for compression tests).

For the characterization of tensile properties, bar-shaped specimens (cross-section: 10 mm × 2 mm ca.) were tested with a gauge length of 40 mm and a crosshead speed of 0.5 mm, until failure. Tensile modulus (measured as the initial slope of stress–strain curves), stress at break and strain at break were evaluated.

For compression tests, cylindrical specimens (diameter: 10 mm ca.; height: 12 mm ca.) were deformed between two parallel plates at a strain rate of 10%/min, until 50% strain. The compressive modulus (measured as the initial slope of stress–strain curves) and the stress at 50% strain were evaluated.

Finally, cyclic compression and stress relaxation tests were carried out on cylindrical specimens by applying the following: (i) pre-load of 0.01 N (to establish contact between the specimen and the upper plate); (ii) 10 loading–unloading cycles between 0% and 50% strain at a strain rate of 10%/min (serving as preconditioning, to stabilize the material mechanical behavior); (iii) stress relaxation at 15% strain, quickly applied at 60 mm/min and maintained constant for 15 min. The dissipation energy of the hydrogels per unit volume was determined by calculating the hysteresis loop area for each loading–unloading cycle, according to the following equation:

(5) $$Dissipation\;energy\;\left[\mathrm{kJ}/m^{3}\right]=\int_{0}^{\varepsilon_{max}}\sigma^{+}d\varepsilon +\int_{\varepsilon_{max}}^{0}\sigma^{-}d\varepsilon$$

where $\varepsilon_{max}$ is the maximum strain, $\sigma^{+}$ refers to the stress during loading, and $\sigma^{-}$ refers to the stress during unloading. The dissipation energy was also evaluated as percentage with respect to the area under the loading curve, as follows:

(6) $$Percentage\;dissipation\;energy\;\left[\%\right]=\frac{\int_{0}^{\varepsilon_{max}}\sigma^{+}d\varepsilon +\int_{\varepsilon_{max}}^{0}\sigma^{-}d\varepsilon }{\int_{0}^{\varepsilon_{max}}\sigma^{+}d\varepsilon }\times 100$$

The stress relaxation behavior was evaluated in terms of reduced relaxation function [36], by normalizing the stress with respect to $\sigma_{max}$ (i.e., the maximum stress reached when applying 15% strain) and plotting it against time, which resulted in a decreasing trend until reaching a plateau stress ($\sigma_{plateau}$). The stress relaxation was calculated according to the following equation:

(7) $$\mathrm{Stress}\;\mathrm{relaxation}\;\left[\%\right]=\left(1-\frac{\sigma_{plateau}}{\sigma_{max}}\right)\times 100$$

where $\sigma_{plateau}$ is the stress value associated with the plateau. Moreover, the half stress-relaxation time ($t_{1/2}$) was defined as the time by which half of the total stress relaxation was achieved.

### 4.6. In Vitro Biological Characterization

#### 4.6.1. Human Umbilical Cord-Derived Mesenchymal Stem Cells Culture

Human Umbilical Cord-derived Mesenchymal Stem Cells (hUC-MSCs) were purchased from PromoCell, Germany. hUC-MSCs were expanded in flask at a density of 3000 cells/cm^2^ in Dulbecco’s modified Eagle’s medium (DMEM), a high glucose-based medium with 1% penicillin-streptomycin, L-glutamine, sodium pyruvate, MEM Non Essential Amino Acids solution, 0.05% amphotericin B (stock solution, 10,000 U/mL penicillin, 10 mg/mL streptomycin, 200 mM l-glutamine, 100 mM sodium pyruvate, 100X MEM Non Essential Amino Acids solution, 250 μg/mL amphotericin B) and 10% fetal bovine serum (FBS), and cultivated at 37 °C, 5% CO_2_ in a humidified atmosphere. For the following experiments, a complete medium, as described above, supplemented with 5% hPL instead of FBS, was employed.

#### 4.6.2. Human Platelet Lysate Production

Human Platelet Lysate (hPL) for MSCs expansion was obtained from blood donations belonging to the Blood Bank of ASST Spedali Civili of Brescia, Italy and produced according to standardized clinical grade procedures in closed systems [45] and as previously described [21].

#### 4.6.3. hUC-MSCs Cell Proliferation and Cell Viability Assay

hUC-MSCs were detached from the flask using 0.25% trypsin ethylenediaminetetraacetic acid after reaching 80% of confluence, neutralized with complete medium +10% FBS, centrifuged at 1100 rpm for 5 min, resuspended in 1 mL of basal growth medium and counted with an automated cell counter (NanoEntek, Inc, Seoul, South Korea). Then, hUC-MSCs were seeded at a cellular density of 3000 cells/mm^3^ (48 × 10^4^ cells/scaffold) into the two different types of scaffolds: G-PEG-CARR and G-PEG-CARR-CH. Scaffolds with volume of approximately 160 mm^3^ were used. The required amount of cells was resuspended with complete medium +5% hPL and added to each scaffold by direct cell seeding in 24 uncoated multiwell plates, then incubated at 37 °C under 5% of CO_2_ conditions for 30 min. After this time, 1 mL of complete medium +5% hPL was added to each well.

Cell viability in the scaffolds was assessed using Live/Dead kit for mammalian cells (ThermoFisher, Norristown, PA, USA) at days 3 and 21 of 3D culture. The samples were washed twice with Dulbecco’s phosphate-buffered saline (DPBS) and incubated for 30–45 min at room temperature in DPBS with 2 μM of calcein AM and 4 μM of ethidium homodimer-1 (EthD-1). NucBlue^®^ Live reagent (2 drops/mL) for nuclei staining was added to the samples. A qualitative analysis of live (stained in green with calcein AM) and dead (stained in red with EthD-1) cells was then performed using Olympus IX83 and Olympus IX70 fluorescence microscopes.

Cell proliferation was evaluated using the Cell-Counting Kit-8 (CCK-8, Sigma-Aldrich, St. Louis, MO, USA) after 2, 7, 14, and 21 days of culture, following manufacturer instructions. Briefly, the cell-seeded scaffolds were moved to a new cell culture plate at each time point and incubated with fresh culture medium +5% hPL containing the CCK-8 reagent (ratio 1:10) at 37 °C under 5% of CO_2_ for 2 h. The samples were placed into another cell culture plate and were twice washed with DPBS. The absorbance of 100 μL of supernatant transferred to a 96 uncoated multiwell plate was measured at 450 nm using a Tecan Infinite 200 spectrophotometer (Tecan Group Ltd., Männedorf, Switzerland). Absorbance at 450 nm is proportional to the number of viable cells in each sample. In fact, a standard curve was generated by cultivating a known quantity of viable cells in order to calculate the relative amount of viable cells proliferating into the scaffolds.

#### 4.6.4. Statistical Analysis

For the statistical analysis of the in vitro assessment of cell proliferation on the scaffolds GraphPad Prism software (version 8) was used. Two-way ANOVA with Tukey’s post hoc test or unpaired *t*-test was performed as specified in the captions in the Figures. Three replicates of each sample were used for biological assays. Statistical significance was accepted at the probability level *p* < 0.05. Data are expressed as mean ± standard deviation.

## Figures and Tables

**Table 1:** Composition and physical properties of G-PEG-CARR and G-PEG-CARR-CH hydrogels.

Hydrogel	Composition				Physical Properties		
	G [wt%]	PEG [wt%]	CARR [wt%]	CH [wt%]	Apparent Density [g/cm^3^]	Porosity [%]	Swelling Ratio (24 h) [%]
G-PEG-CARR	75	17.5	7.5	-	0.074 ± 0.01	80 ± 5	550 ± 15
G-PEG-CARR-CH	69	17	6	8	0.080 ± 0.01	78 ± 7	830 ± 25

**Table 2:** Tensile and compressive properties of G-PEG-CARR and G-PEG-CARR-CH hydrogels after immersion in water for 24 h.

Hydrogel	Tensile Properties			Compressive Properties	
	Modulus [kPa]	Stress at Break [kPa]	Strain at Break [%]	Modulus [kPa]	Stress at 50% [kPa]
G-PEG-CARR	1400 ± 200	130 ± 20	11 ± 2	31 ± 6	11 ± 2
G-PEG-CARR-CH	140 ± 20	36 ± 5	26 ± 9	63 ± 10	16 ± 2

## Data Availability

The data presented in this study are openly available in article.
